# The design of mapping populations: Impacts of geographic scale on genetic architecture and mapping efficacy for defense and immunity

**DOI:** 10.1016/j.pbi.2023.102399

**Published:** 2023-08

**Authors:** Andrew D. Gloss, Margaret C. Steiner, John Novembre, Joy Bergelson

**Affiliations:** 1 Department of Biology, Center for Genomics and Systems Biology, New York University, New York, NY, USA; 2 Department of Human Genetics, University of Chicago, Chicago, IL, USA; 3 Department of Ecology & Evolution, University of Chicago, Chicago, IL, USA

## Abstract

Genome-wide association studies (GWAS) have yielded tremendous insight into the genetic architecture of trait variation. However, the collections of loci they uncover are far from exhaustive. As many of the complicating factors that confound or limit the efficacy of GWAS are exaggerated over broad geographic scales, a shift toward more analyses using mapping panels sampled from narrow geographic localities (“local” populations) could provide novel, complementary insights. Here, we present an overview of the major complicating factors, review mounting evidence from genomic analyses that these factors are pervasive, and synthesize theoretical and empirical evidence for the power of GWAS in local populations.

## Introduction

Genome-wide association studies (GWAS) have spurred advances in breeding high-yield and stress-resistant crops [1,2], uncovering adaptive loci in nature [3], and understanding and treating human disease [4]. Yet, GWAS typically reveal only a fraction of the genetic variants affecting a trait, and these associated loci often fail to explain the majority of heritable trait variation [5].

Ironically, one of the greatest allures of GWAS may underlie this shortcoming. Unlike genetic mapping using offspring from an experimental cross, in which only genetic variants present in the parents are interrogated, GWAS mapping panels capture deep pools of genetic variation from natural populations. At the same time, however, the complexities of how these alleles are assorted across individuals and collectively shape phenotypes are not necessarily captured well by the standard GWAS model (Box 1) [4,6].

Box 1 **The standard GWAS model.**

Most often, GWAS employs linear regression to test for the effect of a SNP genotype on a trait. This can be expressed as **Y** = **Xβ** + **g + e**, where **Y** is a vector of phenotype values across individuals, **X** is each individual's genotype at the focal SNP, **β** is the phenotypic effect of that SNP, **g** is the collective polygenic effect of other SNPs, and **e** is residual error [60]. In linear mixed models, **g** can be captured by including a matrix of genetic relatedness (kinship) among individuals as a random effect; this is a preferred approach to account for population structure [9].

The flexibility of linear regression lends itself to extensions that incorporate covariates, multiple loci, environment and gene-by-environment effects, pleiotropy among traits, and epistasis—though computational complexity and multiple testing burdens may limit their feasibility or power. However, the standard GWAS model also suffers from the usual limitations of linear regression, such as limited power when there are few data points for levels of a predictor variable (e.g., only a few individuals with the less common SNP genotype in the mapping panel).

Alt-text: Box 1

**Figure 1:**
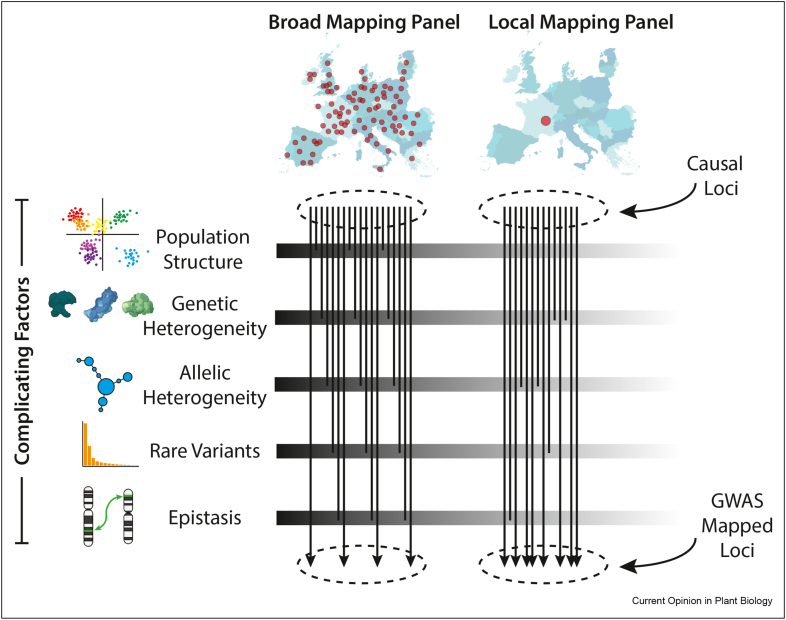
**Expected effects of the geographic scale of the mapping panel on GWAS efficacy**. Although geographically broad populations are expected to harbor more genetic diversity and thus more loci segregating for causal variants, many factors that complicate GWAS are worsened over broad geographic scales. These factors may cause fewer associated loci to be discovered by GWAS than in narrowly sampled populations. Note that this is meant to be a conceptual illustration only. Disparities in the number of causal loci – and the relative impacts of each complicating factor – will differ across traits and study organisms due to demography, selection, and biological properties of the focal trait, among other factors.

Here, we argue that by mapping in individuals derived from a narrow geographic locality (“local” mapping populations), more efficient discovery of significant associations is possible due to a reduction in sources of complexity that confound or complicate GWAS. First, we introduce these sources of complexity and explain how they are affected by the geographic composition of the mapping panel (Figure 1). Second, we review recent studies that demonstrate the benefits of local mapping populations, drawing on GWAS in both plants and humans. Finally, we discuss limitations of local mapping populations, and highlight key areas for future research. Our exploration of these concepts will aid geneticists in considering the effects of mapping population design when seeking a more complete catalog of the genes shaping phenotypic variation in nature.

## Factors that complicate GWAS across geographic scales

### Population structure

Of the factors that confound GWAS, the most infamous is population structure. This arises when a population has sub-groups between which mating is restricted, often due to geographic barriers or distance, causing allele frequencies to vary spatially. Population structure can lead to false-positive GWAS associations because it can lead to correlations between genotypes at causal and non-causal loci, or between causal genotypes and environmental variables that affect the phenotype of interest [7,8]. Although the incorporation of a kinship matrix and/or fixed effects representing genetic ancestry in the GWAS model can control for spurious associations (Box 1) [9], this comes at the cost of reducing power to detect causal variants whose geographic distribution tracks major axes of population structure [10,11]. Furthermore, this approach can fail to fully control for confounding by population structure. Although these subtle, incompletely controlled effects may be too weak to drive significant associations at individual loci, they can be compounded in problematic ways in genome-wide analyses such as polygenic scores, especially in meta-analyses across heterogeneous populations [12,13].

The confounding effects of geographic population structure could be mitigated by choosing a local mapping population. When sampling individuals locally, dispersal distances are larger relative to the sampling range, so geographic variation in allele frequencies tends to be smaller. Thus, the confounding effects of geographic population structure should be weakened.

### Genetic heterogeneity

A major determinant of power in GWAS is the number of loci contributing to a trait with a given amount of additive genetic variation (heritability) [14]. As the number of loci contributing to a trait increases, the variation explained by each locus decreases. Thus, genetic heterogeneity, the phenomenon in which alleles from different loci contribute to the same phenotype [6,15,16], decreases mapping power [17]. Recent analyses suggest genetic heterogeneity is remarkably common in both plants [18,19] and humans [20].

**Figure 2:**
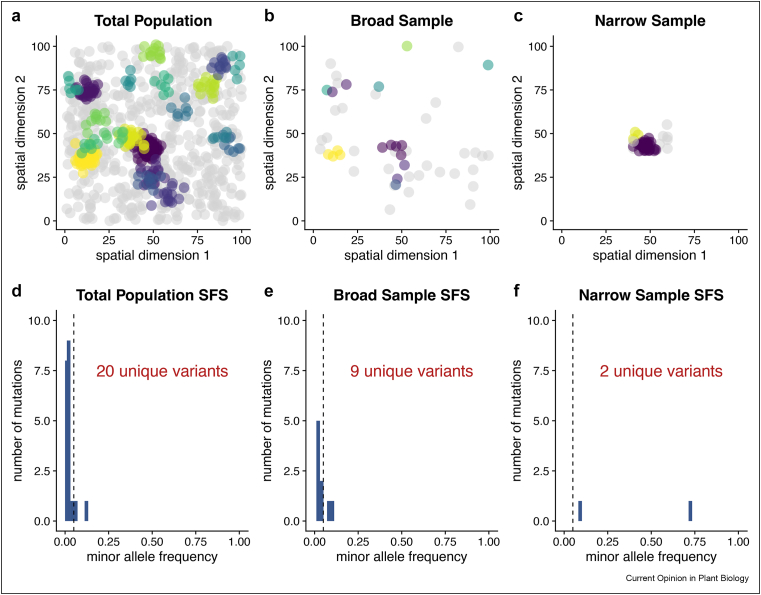
**Illustration of how broad vs. narrow sampling may affect the discovery of derived alleles**. The top row (panels A–C) depicts the geographic distribution of genotypes in a hypothesized scenario in which individuals homozygous for the ancestral allele (grey circles) are widespread and carriers of 20 different derived alleles are geographically clustered (colored circles, with one color per unique allele). The geographic distribution of genotypes is shown for three scenarios: (**a**) the total population (n = 868), (**b**) a sample of the total population based on collection effort that is spread out across the space (“broad sample”, n = 50), and (**c**) a sample based on collection effort that is geographically focused (“narrow sample”, n = 50). The second row (panels D–F) shows the resulting site frequency spectrum (SFS) in each scenario. The vertical dashed line in (D–F) denotes a MAF of 5%, a standard filter applied for mapping studies in plants. In panels B and E, one sees a “broad” sample of 50 individuals sampled uniformly across the entire habitat yielding carriers of nine unique variants (out of 20 total). MAFs within the sample are in a similar range as that of the entire population. In panels C and F, one sees a “narrow” sample of 50 individuals from a smaller region of the habitat yields carriers of only two unique variants, though the MAFs for the variants are relatively high (e.g., one variant reaches nearly 75% in the narrow sample) and all surpass the 5% MAF filter, suggesting the potential effects of narrow sampling on MAF range.

Geography contributes to genetic heterogeneity in two key ways. First, many alleles have geographically restricted distributions [21], causing the genetic basis of a trait to vary across regions [18]. This weakens GWAS signals, because geographically restricted causal variants fail to explain trait variation in regions where they are not segregating. Second, local samples typically contain fewer polymorphic causal loci than samples collected across broader geographic ranges. This is especially true in highly structured populations where founder events and population bottlenecks occur locally, and remains true with continuous population structure [22,23]. Accordingly, the proportion of variation explained by each polymorphic causal locus is expected to be higher in local populations. We illustrate these intuitions in a hypothetical scenario with a geographically structured distribution of causal variants (Figure 2).

Notably, one recent study in *Arabidopsis thaliana* [18] investigated the geography of genetic heterogeneity using expression levels at thousands of genes as individual traits. Cases of different causal variants across geographic regions were observed more than twice as often as shared causal variants across regions, underscoring how broad geographic sampling exacerbates genetic heterogeneity.

### Allelic heterogeneity

Not only are most traits affected by many loci, but many causal loci harbor more than two functionally distinct haplotypes, termed allelic heterogeneity [6,16]. Allelic heterogeneity reduces mapping power because each haplotype explains only a fraction of the variation accounted for by a locus. This is particularly problematic when employing GWAS models that test each biallelic Single Nucleotide Polymorphism (SNP) individually rather than testing across haplotypes at a locus; in the former case, an allele may tag multiple haplotypes, which may have different or even opposing phenotypic effects.

Sampling from a local population is expected to produce less allelic heterogeneity than sampling broadly, as both the exclusion of some geographically restricted alleles and the overall reduction in genetic diversity would lead to fewer alleles per locus. Our illustration of sampling spatially structured causal variants (Figure 2) depicts this when one assumes these are alleles from a single locus.

Recent analyses suggest allelic heterogeneity is common in plants and humans, and could likely be found at most casual loci given sufficient power [19,20,24]. Although developing a systematic, genome-scale understanding of the extent of allelic heterogeneity is an open challenge, anecdotal evidence suggests it may be particularly common for genes involved in defense and immunity. For example, allelic heterogeneity can arise from pressures to recognize different pathogens [25] or to fine-tune investment in defense [26].

### Rare variants

Globally, most alleles are rare (i.e., at low frequency) [21]. Power to detect phenotypic effects of individual rare variants is limited because they are found in few individuals within a mapping panel. Further, the interaction between rare variants and the complicating factors mentioned above poses additional challenges. For example, rare variants tend to be more geographically restricted than common variants [21,27]. Methods to account for environmental or background genetic factors may be less effective for these sharply structured distributions, leading to false positives and obscuring true associations [28]. Finally, the presence of multiple geographically restricted rare alleles can manifest as allelic heterogeneity at the population level. The major locus underlying cystic fibrosis, which segregates many causal variants that are usually rare, geographically structured, and geographically restricted [29], provides a strong illustration of these issues.

Local mapping panels may lessen the impact of each of these factors. First, both simulations and empirical studies have shown that the SFS is shifted toward intermediate frequency variants in local populations, lessening the proportion of variants that are rare [22,23,30, 31, 32] (also see Figure 2). This effect arises in both continuously structured and subdivided populations, and can persist even in the face of relatively high migration rates/large dispersal distances [23,32]. Second, the restricted geographic distribution of globally rare variants implies that each one will be more common in the region(s) where it is present and thus more likely to explain a statistically discoverable portion of phenotypic variance.

### Epistasis

Epistasis refers to differences in a causal variant's effect across genetic backgrounds. GWAS is less powerful when a variant's effect is weakened or reversed in some genetic backgrounds, since standard GWAS models are formulated to detect average additive effects [33,34]. Theory predicts that epistasis should be common in molecular networks and propagate to higher-order phenotypes [35], and this is supported in high-powered QTL mapping studies in yeast [36,37] and more modest studies in plants (e.g., Ref. [38]).

Population structure across multiple causal loci can produce different genotypic combinations in different geographic regions, especially for large effect, locally adaptive loci (e.g., Refs. [39,40]). These large effect loci may be less variable in local populations with less genetic diversity and lacking broad geographic structure, leading to less variable genetic backgrounds and less severe loss of mapping power from epistasis [41].

## Insights from GWAS in local populations

### Case-study: Defense and immunity in *A. thaliana*

Although local mapping panels are uncommon in plants, an *Arabidopsis* population from Toulon-sur-Arroux, France (TOU-A) provides an excellent example of local genetic and phenotypic diversity. Whole-genome sequencing was conducted for 195 accessions collected over a 350 m transect [42]. GWAS uncovered loci shaping quantitative resistance to pathogens [43,44], recognition or response to pathogen effectors [42,44], and production of chemical defenses (glucosinolates) in this population [41]—in addition to growth, phenology, and fitness [42,45]. Dissections of candidate defense and immunity loci in other *Arabidopsis* populations suggest the substantial diversity observed in TOU-A population is unlikely to be unique. Local German populations harbor multiple functionally distinct alleles at a regulator of defense (*ACD6*), beyond what could be discerned from GWAS of a broader population [26]. Similarly, a presence/absence polymorphism of a resistance gene (*RPS5*) segregates at intermediate frequency in most populations globally [46].

A comparison [41] of GWAS for glucosinolates conducted either in TOU-A or broad European panels [39,40] underscores the potential advantages of GWAS in local populations. Although heritability was similar across panels, twice as many biosynthetic loci were associated with variation in these defensive metabolites in TOU-A. Distinct associations for quantitative disease resistance in TOU-A compared to a geographically broad panel further highlight how local populations can reveal novel causal loci [44].

### Case-study: Autoimmunity in humans and focal genetic studies of the Sardinian population

In humans, one major use of local mapping panels has been the study of “population isolates” or “founder populations” [47]. Such populations should exhibit many of the advantages for mapping described here: reduced allelic and genetic heterogeneity, less environmental confounding, and an enrichment of intermediate frequency alleles.

A particularly well-studied local mapping panel is composed of individuals from the Mediterranean island of Sardinia. GWAS in these samples has revealed a number of novel and compelling loci [48, 49, 50, 51]. Several of the loci discovered in GWAS on Sardinia and not the mainland have a higher frequency in Sardinia (e.g., Refs. [52,53]). Further, haplotype diversity patterns at some of these loci suggest a history of recent positive selection in Sardinia as a mechanism for the elevated frequencies, perhaps driven by locally acting pressures such as resistance to malaria.

One fascinating example [53] is the discovery of a risk allele for the autoimmunity condition of multiple sclerosis (MS) through GWAS in Sardinia. The same locus is also a QTL for 1) levels of the “B-cell activating factor” (BAFF) protein in the circulating blood, 2) the transcript abundance of the *TNFSF13B* gene which encodes BAFF protein, 3) the length of the *TNFSF13B* transcript, 4) the number of B cells in the blood. Of note, the MS risk-increasing allele increases B cell levels by truncating the UTR of the transcript and thus removing a microRNA binding site that mediates decay of the transcript and in turn reduces BAFF levels. This mechanism suggests the autoimmunity disorder risk is increased from an overabundance of B cells. Further, the MS risk-increasing allele appears to have been selectively favored based on the haplotype diversity patterns and levels of differentiation with the mainland. While the mechanism of selection is unknown, one might predict that the MS riskincreasing allele provided an adaptive response to past (or ongoing) pathogen exposure by elevating B cell counts.

A more general lesson here is that local mapping populations may reveal novel loci due to past-acting selective pressures that elevated the frequencies of the alleles. This is perhaps especially true for populations where local adaptation is facilitated by periods of isolation where selective pressures at other locations are moderated.

## Limitations of local mapping panels

In many ways, local populations offer some of the advantages of both diverse natural populations and simpler synthetic populations from experimental crosses, while suffering less from some of the drawbacks of each (Box 2). Nevertheless, they are not without limitations. A key consideration is whether local population samples harbor sufficient genetic diversity, especially in cases where genetic drift and founder effects might purge variation from small local populations. Population genetic surveys are therefore a necessary first step to identify suitable local populations, and the ideal geographic scale of a local population may vary among study systems. Even with well-informed sampling, however, genetic variation at some regionally polymorphic causal loci might still be insufficient within a given local population. If feasible, GWAS within more than one local population could avoid this pitfall.

Box 2 **Local populations on the continuum from synthetic mapping panels to global populations.**

Synthetic mapping populations, derived from crosses between a pair or small panel of parents, are a common way to avoid complications that arise in genetically diverse, geographically broad populations. They can eliminate rare variants and geographic population structure, and by including only a subset of the genetic variation in the broader population, they can reduce genetic and allelic heterogeneity [61]. However, they have disadvantages as well: they can be laborious to construct (especially in organisms with long generation times), mapping resolution is limited by the number of recombination events following experimental crosses, and they are limited to the genetic variation present in the parental lines.

Local populations fall in between simplified, synthetic populations and diverse, global populations – offering a balance between the advantages and drawbacks of each. The complicating factors from the previous section are reduced, but not eliminated. And they are already present in nature; most of the labor to “construct” them has already been completed.

Alt-text: Box 2

The utility of local populations may also depend on how selection acting on a trait varies geographically. If selection is sufficiently strong to nearly fix locally adaptive variants, GWAS within local populations will have little power to detect their effects. Meanwhile, mapping populations spanning a broader geographic range (over which these loci are polymorphic) may not only succeed in mapping the effects of these loci on traits and fitness, but also enable population genomic analyses that uncover signatures of local adaptation [39,40]. There are, of course, caveats to these scenarios. Locally adaptive variants whose distributions align with population structure may be difficult to map across broad populations [10]. Some locally adaptive variants may remain globally rare while only reaching a frequency sufficient to detect with GWAS in local populations [54,55]. And local populations are especially effective for inferring how variants conferring fitness tradeoffs are maintained as selection fluctuates within single sites over space and time—particularly when using GWAS to quantify fitness effects of causal variants in different years or habitats [3] or when tracking allele frequency changes in those populations over contemporary timescales [42].

When GWAS are being used as a preliminary step in producing polygenic risk assessment or trait prediction, broad mapping panels will yield higher predictive performance on average across populations [56]. In contrast, effects mapped in a given local population may not be portable to others: effects may be weakened, fail to replicate, or even have opposing effects on the same trait in other populations. This can arise due to differences in linkage disequilibrium between the marker and causal variants across populations, differences in epistatic effects (GxG) when genetic backgrounds vary among populations, and different environmental effects across populations (GxE) [57]. Population-specific biases are a sensitive issue in human genetics, where developing equitable precision medicine is an important goal yet many ethnicities have been vastly underrepresented in GWAS panels [57,58]. Of course, for loci at which discordant effects across populations are pervasive, their additive effects may be weaker (and harder to detect) in broad mapping panels, and studies of local populations individually may be a more powerful and accurate alternative.

Finally, local populations may harbor elevated linkage disequilibrium due to younger coalescence time among individuals [22], and this would be expected to limit mapping resolution in GWAS. In broader panels, the observation of a given causal variant on different haplotypes in different geographic regions breaks down correlations in genotype between the causal variant and nearby non-causal variants [57]. Indeed, fine mapping approaches to infer putatively causal variants within GWAS peaks achieved tighter resolution with a human mapping panel distributed more broadly and evenly across the globe [56]. We note, however, that GWAS within local populations have nonetheless achieved mapping resolution sufficient to identify individual candidate genes [41,43] and causal variants [55].

Overall, local and geographically broad mapping panels are complementary, as they typically reveal different loci associated with a trait (e.g., Ref. [44]), and choices between the two should be motivated by the goals of the study. Given the historically limited use of local mapping panels, however, significant benefits could be gained by more fully embracing their use.

## Conclusions and future outlook

Although most efforts toward mapping phenotypic variation with GWAS have focused on regional or species-wide mapping populations, studies of local populations have been promising. In plants, they've revealed substantial genetic variation that shapes growth, phenology, defense, and immunity—likely maintained, in part, by spatially and temporally heterogeneous selective pressures. Importantly, genetic mapping of these traits has revealed loci that were missed in broader GWAS populations.

Empirical studies of hierarchically constructed mapping panels—with similar numbers of individuals (and thus mapping power) at local, regional, and species-wide scales—are needed to more robustly test if and when GWAS recovers more associated loci locally. Studies of simulated populations will also be invaluable, especially for understanding the causes of such patterns and the extent to which they are context dependent. The contributions of the complicating factors highlighted in this review, their interactions [16], and their interplay with both demography and selection [59] are difficult to tease apart in natural populations, but they can be experimented with in simulations. Indeed, simulations have already begun to demonstrate that the magnitude of local sampling effects on patterns of genetic variation depends on the spatial structure and dispersal, demographic history, and overall genetic diversity of a species [22,23,30,32], with corroborating empirical support from a case study in two recently diverged plant species that differ in each of these properties [30]. There is also a role for theory: as theoretical population genetics often focuses on the case of a well-mixed (panmictic) population, the expected interaction between population structure and sampling remains underexplored. Further modeling which accounts for the joint effects of population structure and geographic breadth of sampling would help to provide insight into expected outcomes.

A deeper understanding of these patterns and mechanisms is already within reach owing to the powerful computational, statistical, high-throughput sequencing and phenotyping resources available. As these studies unfold, they will inform optimal approaches for understanding the genetic architecture and maintenance of trait variation in nature.

## Declaration of competing interest

The authors declare that they have no known competing financial interests or personal relationships that could have appeared to influence the work reported in this paper.

## Data Availability

No data was used for the research described in the article.
